# Sustainability communication on social media in the last decade: A review of research perspectives from Asia

**DOI:** 10.12688/f1000research.159108.1

**Published:** 2024-12-24

**Authors:** Shruthi V Shetty, Smitha Nayak

**Affiliations:** 1 Manipal Institute of Communication, Manipal Academy of Higher Education, Manipal, Karnataka, 576104, India; 2 Department of Humanities and Management, Manipal Institute of Technology, Manipal Academy of Higher Education, Manipal, Karnataka, 576104, India

**Keywords:** sustainability communication, social media, pro-environmental behavior, green marketing, user-generated content, scoping review

## Abstract

The past decade has been marked by increased discussions on sustainability; some of the prominent accelerators being, mounting natural disasters, vocal climate activism, and the UN Agenda 2030 for Sustainable Development. The interest has crossed over from deliberations in global summits to grassroots-level communication. Additionally, the literature points to the increasing usage of social media in the communication of sustainability with an increase of interest in this area among Asian researchers. This paper aims to understand the direction of sustainability communication and the usage of social media in the communication of sustainability, in the last decade (2013-2023), from an Asian perspective – researchers and respondents from Asia. A comprehensive search of the literature by Asian researchers on sustainability communication on social media in the past decade was conducted using the Scopus database. The studies were clustered around common directions and terminologies, basis an in-depth content analysis. Based on the analysis, the study presents prominent themes and the current direction of research in sustainability communication on social media. The studies analyzed in the scoping review affirm that sustainability communication on social media in Asia has substantial potential to improve the environmental awareness of the public and promote actual pro-environmental behavior both in terms of green purchase as well as conservation behavior. The review provides a holistic view of the recent trends and possible future directions for sustainability communication on social media.

## 1. Introduction

Globally, there is a growing awareness about, the imminent catastrophe due to environmental degradation as well as the steps necessary to safeguard ecology ( Camilleri, 2017). A societal transformation is required to counter the environmental damage and to achieve sustainable development goals. While it is true that macro-level changes such as socio-political, technological, and economic are required; a difference can also be made by undertaking the micro-level level change in behavior by individuals who consume materials and energy every day.

Media, communication, and framing of messages have the potential to impact values and lead to behavioral changes ( Evans et al., 2022; Mobeen et al., 2022). This is true for marketing messages that are often blamed for pushing consumers towards materialism as well as social messages that have helped build awareness from Polio in India to COVID-19, globally. It can be argued that a similar effect of communication can help lead society towards a sustainable future. While communication by itself may be insufficient in causing behavioral change ( Siegel et al., 2018), to generate public engagement and initiate policy change, issues like sustainability need to be communicated in the right manner ( Swenson & Olsen, 2017). Evidence suggests a positive correlation between positive messages on media and pro-environmental behavior (PEB) ( Yang et al., 2020).

Sustainability communication originates from environmental communication ( Fischer et al. 2016). Sustainability communication draws from a broad field of different scientific disciplines such as psychology, sociology, media theory, and communication theory. It comprises themes related to climate, energy, consumption, conservation, and corporate sustainability communication ( Voci & Matthias Karmasin, 2023; Sheehy & Farneti, 2021). Sustainability communication by organizations has increased owing to the growing stakeholder demands; in the last decade, companies have become more invested in being vocal and visible about their pro-environment stance ( Bhardwaj et al., 2023).

Brand or corporate sustainability communication engagement has manifested in the form of sustainability reporting on websites ( Stocker et al., 2020) branded content or brand journalism ( Swenson & Olsen, 2017), green marketing campaigns ( Nadanyiova et al., 2020; Bailey et al., 2018) as well as information campaigns ( Tavri, 2019). Organizations today increasingly prefer social media for communicating about sustainability to their stakeholders ( Gupta et al., 2021; Camilleri, 2017). Social media characterizes two-way communication as against the traditional media, this accentuates its role in influencing behavioral change ( Gupta et al., 2021; Loureiro & Lopes, 2019). The users are also increasingly connecting with their preferred brands on social media to engage with sustainability-related information ( Loureiro & Lopes, 2019). Independent social media influencers are engaging with the younger audience (Millennials and Gen Z) in sustainability-related dialogues in fashion, food, and travel ( Kapoor et al., 2021, 2022a).

Culture and values play a significant role in how sustainability communication is perceived. A difference is observed between individualistic cultures and collectivist cultures and, the subsequent effect on message response ( Yang et al., 2020; Paul et al., 2016). Individualistic cultures such as the West, are driven by self-interest, hence climate-related messages with a threat component work well for behavior change. In comparison, collectivist cultures such as in Asian countries, respond to efficacy-related messages better due to the prominence given to social influence ( Han & Cheng, 2020).

Several studies on sustainability, communication of sustainability, and the role played by the media environment in pro-environmental behavior, have emerged from Asia in the past. Researchers have analyzed various facets of media effect, both traditional and new media; media exposure and attention on PEB ( Lee & Cho, 2020), media’s influence on causal variables such as effects of attention towards types of environmental content ( Yang et al., 2020), message appeal and source of the message ( Kapoor et al., 2021), media usage ( Huang, 2016), presumed media influence on others ( Ho et al., 2014) media dependency ( Ho et al., 2014) and media exposure ( Lee, 2011). Social media has emerged as an important platform due to its usage among millennials and Gen Z ( Yamane & Kaneko, 2021; Han & Cheng, 2020; Xu & Han, 2019).

It can be argued that there is a higher quantum of research from developed economies ( Chwialkowska et al., 2020) but studies from non-western countries such as Asia are steadily increasing and adding to the multi-dimensional discourse.

Asian countries collectively are the largest contributors to global emissions ( Wahyuningrum et al., 2023), this makes Asia the epicenter of climate change impact. As per a 2021 McKinsey report $4.7 trillion of the Asian GDP is at risk due to climate change-related issues. The data provided by Kantar’s Asia Sustainability Foundational Study 2021 shows that 58% of Asian consumers are personally affected by environmental problems; 53% of Asians have stopped buying products and services that harm the environment and society, highlighting an opportunity for Asians to take the lead in mitigation. It is pertinent to understand how individuals from Asian countries depend on social media to seek information related to sustainability and how this consecutively influences their behavior ( Saeed et al., 2019). A scoping review of sustainability communication on social media in Asia, would present a more holistic perspective and help future researchers build on this premise. This scoping review aims to achieve the following goals: To map the landscape of research on sustainability communication on social media in Asia over the past decade. Additionally, utilizing the findings to identify research gaps for further evaluation.

Thus, with a unique focus on sustainability communication on social media, this research article attempts to undertake a comprehensive review of all eligible empirical papers that have been published in the past decade. This study attempts to document the implications of the current research paradigms on sustainability communication on social media and identify the directions for future research. Approval for the study was received from the Doctoral Advisory Committee, Manipal Institute of Management, MAHE on March 10, 2022. This marketing study was exempt from submission to the Institutional Ethics Committee of Manipal Academy of Higher Education, by clause 6 of the circular titled “Project Exemption from Submission to IEC,” issued by the Institutional Ethics Committee on 14/01/2021. Subject to this circular, approval is obtained from the MAHE PhD protocol committee (MAHE/CDS/PHD/2022) dated March 31, 2022.

## 2. Method

### 2.1 Search strategy

A comprehensive search and analysis of literature on sustainability communication on social media was conducted in May 2023. The search was conducted in the Scopus database, as high-impact journal articles are indexed here. The focus was Asian studies (respondents and researchers) in the past decade (2013-2023), a decade marked by increased discussions on sustainability. Additionally, Asian countries are seen as taking the sustainability discourse beyond the Western perspective. Focusing on Asian studies in the past decade also gives the current study a more defined focus.

### 2.2 Eligibility criteria

The key inclusion criteria were studies from Asia that focused on Asian respondents and insights, in the past decade. The additional inclusion criteria were journal articles, full text, and those available in the English language. The exclusion criteria were studies that had respondents from non-Asian countries and studies that focused on non-social media platforms such as sustainability communication on websites or applications.

### 2.3 Selection of studies

The first phase of the search included all keywords that were synonyms for the term sustainability communication which yielded 321 documents. The second phase of the search included all keywords that were synonyms for social media and the different platforms of social media which yielded 17,942 documents. An advanced search strategy was used to combine the two searches and filter articles. The keywords used for the search were *(“sustainability communication”, “sustainable communication”, “green communication”, “environmental communication”, “corporate sustainability communication”, “climate change communication”, “pro-environmental communication”, “ecological communication”, “sustainable consumption communication”, “sustainable product communication”, “environment information”, “pro-environment information”, “green consumption communication”, “green advertising”) AND (“social media”, SNS, “social networking sites”, “Twitter”, “Facebook”, “Instagram”, “YouTube”, “WhatsApp”).*

**Figure 1.**
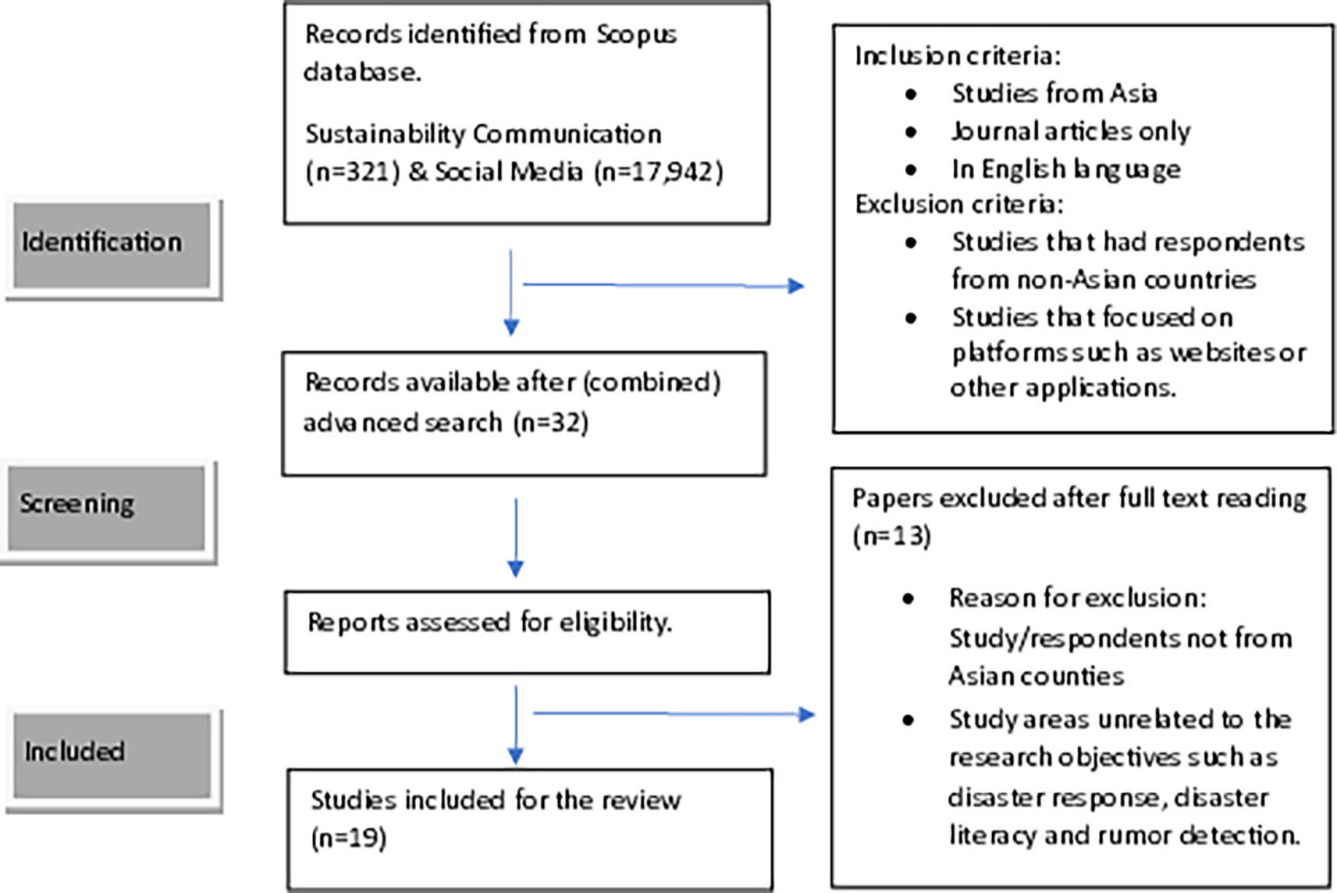
Flow diagram of study selection.

The search yielded 32 documents. Two authors reviewed the studies independently, disagreements on selection were resolved after mutual discussion. The full text of these articles was assessed and evaluated against the eligibility criteria. Studies focusing on areas unrelated to the research objectives, such as disaster response, disaster literacy, and rumor detection, were further eliminated. The final list had 19 research studies that met the eligibility criteria and were included in the review. The flow diagram of the study selection process is presented in Figure 1.

### 2.4 Data extraction

An in-depth content analysis was performed to examine the literature. Data from the 19 papers was extracted and entered on a pre-determined data extraction sheet. Study particulars recorded include author name, year of publication, country, study objectives, study design, key findings, and directions for future research. The summary of articles included in the study is presented in Table 1.

**Table 1.** Summary of articles included in the study.

Si No.	Author & Year	Country	Objectives of the study	Study Design	Findings of the study	Directions for future research
1	Kwon et al., 2023	-	To identify the current trend in green advertising and investigate the prevalence of misleading strategies in green advertising practice in terms of execution context and claim context.	Quantitative Content Analysis	Increasing environmental concerns among consumers have led to additional efforts among corporations to promote their environmental practices. More claims in green advertising on social media were misleading than acceptable. environmental efforts.	Explore different types of social media platforms (e.g., Twitter, YouTube) to provide a more comprehensive understanding of green advertising on social media. Future studies should conduct cross-national content analyses.
2	Rosenthal, 2022	Singapore	To investigate the correlation between perceived personal experience of climate change and different information sources (traditional & social media, interpersonal & institutional sources).	Survey	Perceived personal experience of climate change was associated with the use of traditional media social media & interpersonal sources but not institutional sources.	Not mentioned
3	Noviyanti et al., 2022	Indonesia	To analyze the use of Instagram for environmental communication of social cause.	Qualitative Content Analysis	Verbal and visual campaigns on Instagram proved effective in sharing environmental knowledge. Collaborations are done via action-oriented hashtags.	An empirical study to examine audience awareness and behavioral change post-exposure to social media sustainability-related campaigns.
4	Briandana & Mohamad Saleh,2022	Indonesia	To analyze environmental communication strategies on social media used by environmental NGOs	In-depth interviews	The use of social media (Instagram and Facebook) proved effective in delivering clear messages for social influence.	Use quantitative approaches such as surveys for better feedback on campaign efficiency.
5	Kapoor et al., 2022b	India	To investigate the effectiveness of sustainability messages by green influencers in affecting purchase intention for sustainable products.	Experiment	Sustainability messages by green influencers that have a concrete appeal rather than an abstract appeal are more efficacious as a determinant of the actual behavior among social media users.	Due to the differences in engagement on different social media platforms, it is necessary to assess platform characteristics to understand the efficiency of sustainability messages posted by influencers on social media.
6	Yang, 2022	China	To examine the role of non-English user-generated content on social media, in climate change discussion.	Online Ethnography	Social media promotes participatory culture in the form of citizen science communicators and facilitates audience empowerment.	Not mentioned
7	Chai et al., 2022	Malaysia	To investigate the awareness of university students towards environmental information presented online including types and issue presentation.	Online surveys & in-depth interviews	A modest fit was found between the awareness of students and environmental communication on social media. Visual communication was found to be most effective; Facebook and YouTube were identified as high-exposure platforms.	Green practices among university students to be assessed.
8	Li et al., 2022	China	To examine green advertising on social media and the influence of celebrities in the promotion of sustainability.	Experiment	Consumers’ perception of both brand and celebrity altruism is higher when social media advertisement is organic rather than sponsored.	Use online field experiments to directly observe the social media users’ actual behavior. Can replicate the experiment on other social media platforms.
9	Wei et al., 2021	China	To investigate the impact of an organization’s CSR communication through social media on consumer loyalty with e-WOM as the mediator.	Survey	A direct relationship is seen between CSR communication on social media and loyalty with e-WOM as a potential mediator.	Include consumers’ PEB and organizational environmental commitment in the framework to better perceive consumer behavior.
10	Ch et al., 2021	Indonesia	To examine the role of green marketing on social media in shaping green purchase behavior.	Survey	Significant influence of eco-labeling and green advertising on the decision to purchase green products found.	Focus on a bigger sample from non-metropolitan cities and study respondents with limited knowledge of green products, to get different perspectives.
11	Wang et al., 2021	China, Singapore & South Korea	To analyze the shipping industry’s sustainability communications on social media-Twitter	Content Analysis	There is a direct relationship between CSR communication on social media and consumer loyalty with e-WOM as a potential mediator.	To examine if the emotional appeal used in organizational CSR communication on social media generates brand equity.
12	León et al., 2021	Kuwait	To explore strategies used by advocacy institutions to communicate climate change on social media.	Semi-structured Interviews	Strategies focus more on sender–receiver models of communication rather than looking at social media as virtual meeting spaces for discussion on the environment.	Possible to analyze the effectiveness of the mentioned strategies in promoting citizen participation in climate change discussion.
13	Cao et al., 2021	China	To examine if the interactive nature of social media influences green purchase.	Survey	Green products advertised through highly social advertisements are preferred. Consumers’ green involvement acts as a moderator.	Investigate audience perceptions of green ads in diverse media environments. Compare consumer purchase intentions across varied green products.
14	Kong et al., 2020	South Korea	To understand the interconnection of sustainability communication and e-WOM and purchase intentions (South Korea and Germany).	Experiment	Sustainability advertising on social media is most effective (in terms of its influence on e-WOM and purchase intention) for non-luxury brands and among consumers who have a higher awareness of sustainability.	To explore the influence of cognition and its effect on the processing of sustainability communication.
15	Chen, 2020	China	To understand the factors motivating the sharing of environmental information in the Chinese context.	Survey	Entertainment, presentation of oneself, and having the opportunity to interact with others have a positive influence on information sharing. Awareness of the consequences also has an impact.	Demographic differences can be considered (due to their influence on PEB & social media usage) to create more effective communication strategies.
16	Shafqat & Fong, 2020	Malaysia	To explore the role of climate change communication by environmental NGOs in fostering PEB.	Content analysis and expert interviews	The information-oriented posts and posts on organizational events elicited higher engagement compared to community-building posts.	Explore both drivers and barriers to PEB in Malaysia. Also understand the varied levels of audience engagement with sustainability-related content, online.
17	Luo et al., 2020	China	To analyze the effect of skepticism towards social media green advertising on information utility and purchase intention.	Survey	Skepticism towards green advertising indirectly affects green purchase intention by mediating the perception of information utility.	Undertake cross-cultural research. Also, explore the effect of eco-labeling on green purchase behavior.
18	Sulistyawati et al., 2019	Indonesia	To explore the utility of social media (Facebook and Instagram) and websites to increase environmental awareness among teenagers.	Content Analysis	Instagram emerged as the preferred platform for climate change-related information sharing among teenagers.	Not mentioned
19	Jiang et al., 2018	China	To examine the key influencing factors behind climate change discussions by analyzing user-generated content (UGC) on social media.	Content Analysis	The UGC focus is more on human/societal issues than climate change as a natural phenomenon. Higher engagement is seen for posts on specific subjects, usage of scientific terms, and posts by people with a higher number of followers.	Reduction of subjectivity by using more automated topic modeling approaches.

## 3. Data analysis

A detailed analysis of the 19 studies revealed several insights. The theoretical frameworks used, the direction of sustainability communication on social media in Asia, and the prominent themes that emerged are discussed.

## 4. Results

### 4.1 Theoretical underpinnings

The theoretical underpinnings of the analyzed studies were heterogeneous with two theories mentioned in higher frequency. Azjen’s Theory of Planned Behaviour (TPB) is largely used by researchers to predict pro-environmental behavior; the core components of subjective norms, perceived behavioral control, and specifically attitude in varying degrees correlate to environmentally significant behavior ( Yang et al., 2020; Paul et al., 2016; Ho et al., 2014). The other influential model is the Value-Belief-Norm (VBN) theory by Stern. The VBN theory proposes a framework to hypothesize the effect of environmental values on environmental attitudes. According to this theory, three different value orientations guide individual attitudes: concern for the natural environment - biospheric, interest in the wellbeing of all human beings – altruistic, and self-interest – egoism ( Huang, 2016; Chen, 2014). With increasing green consumers, green consumption value – exploring environmental protection through one’s consumption behavior, is gaining significance and affecting trust towards green marketing communication ( Bailey et al., 2018).

The other theories used include Heider’s Attribution Theory and Gouldner’s Theory of Norm Reciprocity ( Wei et al., 2021), Bateson’s Framing Theory ( Kwon et al., 2023), Blumler and Katz’s Uses and Gratification Theory ( Chen, 2020), Schwartz’s Norm Activation Model ( Rosenthal, 2022), Elaboration Likelihood Model by Petty et al., and Theory of Social Presence by Short et al. ( Cao et al., 2021).

### 4.2 Direction of sustainability communication on social media in Asia

The direction of research identifies prominent entities using sustainability communication in Asia. Studies examine brand-generated sustainability communication on social media focused on purchase intent and building customer loyalty ( Kapoor et al., 2022b, 2023; Luo et al., 2020). This is followed by cause-generated content by environmental non-governmental organizations (ENGOs), for building awareness in the community and fostering positive behavioral change ( Rosenthal, 2022; León et al., 2021). Studies also focus on user-generated content for information sharing and knowledge transfer of sustainability-related topics by the public or the non-scientific community ( Yang, 2022; Chen, 2020). Finally, researchers analyze the awareness regarding online environmental information among adolescents and university students ( Chai et al., 2022; Sulistyawati et al., 2019).

4.2.1 Sustainability communication by marketers/brands

Multiple factors such as eco-labeling, awareness of environmental issues, green advertisements, and price are identified to be the motivators for young buyers to make green purchase decisions ( Ch et al., 2021). Corporations respond to the increased environmental interest of the customers by enhancing their environmental efforts. While the quantum of sustainability communication especially in the form of green advertising has doubled over the years, some of the green claims made by brands on social media may be misleading ( Kwon et al., 2023). Another viewpoint is to look at its effect on purchase; consumers are more likely to purchase green products when they perceive green advertisements to be providing useful information ( Luo et al., 2020).

Consumers are receptive in varying degrees to highly social green advertising (newsfeed advertisements on social media), this in turn influences their receptiveness to new green products ( Cao et al., 2021). Effective use of social media for green advertising is critical to building the pro-sustainability image of the company ( Li et al., 2022). One way is the selection of celebrities to convey sustainability commitment and the mode of message delivery. The results show that customers perceived greater altruism toward brands when the level of similarity between product and celebrity was high; additionally, the post was organic rather than paid.

Analysis of companies’ use of Twitter to convey environmental commitment shows a positive response, with the public being able to discern the industry’s contribution towards sustainability ( Wang et al., 2021). Similarly, the examination of organization’s CSR-related communication on social media, points to a direct relation between CSR communication and customer loyalty, with e-WOM being a potential mediator ( Wei et al., 2021).

External factors impact the response towards sustainability communication. European consumers were influenced more by sustainability communication compared to consumers from Asia. Additionally, non-luxury brands seem to benefit more from association with sustainability compared to luxury brands ( Kong et al., 2020). In the area of message content, sustainability messages by green influencers, with concrete appeal (compared to abstract appeal) were found to be more effective in influencing social media users’ intention to buy sustainable products ( Kapoor et al., 2022b).

Interviews with communication directors and community managers in several countries reveal climate change communication strategies that range from promoting environmental awareness among citizens (general benefit), to community development for the benefit of the organization ( León et al., 2021). Further, these organizations prefer the sender-receiver communication model instead of the more effective dialogue model that facilitates the participation of key audiences.

4.2.2 Sustainability communication by ENGOs and causes

An inter-relationship between climate change beliefs, personal experience (perceived), and different sources of information (social media, traditional media, and interpersonal sources) is possible. In the absence of direct experience, people rely on information from media and interpersonal sources to understand and form climate change beliefs ( Rosenthal, 2022). Effective use of social media to relay sustainability-related information can yield positive results for organizations working towards cause awareness, encouraging PEB for community development ( León et al., 2021). Asian researchers are further looking at both these premises in detail.

The social influence exerted by social media, aids in knowledge transfer for ENGOs ( Briandana & Mohamad Saleh, 2022). Sustainability communication on social media is an effective tool to deliver messages for behavioral change among the local community ( Noviyanti et al., 2022; Shafqat & Fong, 2020).

4.2.3 Sustainability communication as user-generated content

User-generated climate change discussion and construction of sustainability-related narratives on social media is another direction taken by research on sustainability communication. Climate change dialogue on online question-and-answer communities such as Quora, points to an increased discussion about scientific research, energy crisis, and societal impact rather than the specifics of climate change, with a higher engagement seen in posts with more images ( Jiang et al., 2018). The primary motive for user-generated content on sustainability and climate change is the gratification of social interaction and self-presentation ( Chen, 2020). Acknowledgment of responsibility and awareness of consequences do have an influence on altruistic motivations for environmental information sharing.

Public engagement with communication related to climate change on knowledge-sharing platforms facilitates a culture of participation and empowering the audience in the form of CSCs-citizen science communicators. The CSCs are found to be more active than the scientific community with a focus on scientific as well as grassroots or non-scientific discourse ( Yang, 2022).

4.2.4 Sustainability communication and youth

An emerging direction in Asian research on sustainability communication is the focus on adolescents’ and university students’ awareness regarding online environmental information and its impact. Communication about climate change among teenagers is more effective on visual platforms such as Instagram ( Sulistyawati et al., 2019). From the perspective of issue presentation and information type, YouTube and Facebook are considered high-exposure platforms for environmental communication due to the higher incidence of visual communication ( Chai et al., 2022).

### 4.3 Utility of social media in sustainability communication

Using social media to understand public opinion on climate change issues is an evolving research agenda ( Jiang et al., 2018). The likes, shares, and comments play a prominent role in active information sharing ( Chen, 2020) and getting immediate feedback ( León et al., 2021). Social media helps reach opinion leaders and policymakers directly and fosters dialogic discussion between experts and the public ( Yang, 2022). It is a virtual meeting place that can foster a feeling of community and engagement ( Rosenthal, 2022).

The increase in conversation around green marketing on social media is evident in the research focus over the past decade. Multiple studies included in the review examine green advertising on social media and its effectiveness in conveying green product-related information ( Ch et al., 2021; Kong et al., 2020; Luo et al., 2020); also, to disseminate the environmental commitment of the company ( Wei et al., 2021; Wang et al., 2021) and its pro-environmental practices ( Kwon et al., 2023). Green newsfeeds and e-WOM have a positive effect not only on immediate green purchases but also on long-term consumer loyalty ( Cao et al., 2021).

While few studies consider social media as a single unit, others stress on the need to look at individual platforms differently in terms of utility and engagement ( Kapoor et al., 2022b). Twitter is the company’s preferred channel to communicate sustainability agenda ( Wang et al., 2021) and CSR activities ( Wei et al., 2021) with the public or specific stakeholder groups.

Facebook communities replicate actual communities and inform members about pro-environmental lifestyles and sustainable behaviors ( Chai et al., 2022; Shafqat & Fong, 2020). Both Facebook and Instagram contribute to the transfer of pro-environmental knowledge and values ( Briandana & Mohamad Saleh, 2022). Instagram which is a visual-first social media platform is effective in conveying climate-centric messages that have an impact on the audience ( Kwon et al., 2023; Sulistyawati et al., 2019). It also facilitates knowledge transfer via visual and verbal campaigns and, actionable hashtags ( Noviyanti et al., 2022). It is interesting to note that organic posts on Instagram have more credibility than company-sponsored posts. This indicates that social media is seen as a place for users to share opinions rather than marketers ( Li et al., 2022). The studies that look at user-generated content, reiterate the perception that social media is primarily for active participation by the audience or users ( Yang, 2022; Jiang et al., 2018).

### 4.4 Prominent themes in sustainability communication on social media

The direction of sustainability communication and usage of social media in this context puts forward a few prominent themes. The five frequent themes that emerged during the analysis are ‘ *green marketing*, ‘ *greenwashing/skepticism towards green marketing’*, ‘ *social media as a platform for active engagement*’, ‘ *value of user-generated content on social media’*, and ‘ *social media for social influence/as an awareness agent’.* The themes identified and their brief overview are presented in Table 2. These themes give an understanding of the key focus areas of Asian researchers working on sustainability communication on social media.

**Table 2.** Overview of the themes from literature in sustainability communication on social media.

Themes	Overview
Green Marketing	Use of green advertising, green influencers, and celebrities to persuade customers to purchase green products-encourage sustainable consumption.
Greenwashing	Misleading claims in green advertising and customer skepticism.
Social media as a platform for active engagement	Promoting participatory culture, active knowledge sharing instead of being a passive audience; creation of a virtual meeting place for discussions and feedback.
Value of user-generated content on social media	A major platform for public participation, users as content creators, can frame issues and influence discussions, a forum for users (non-scientific community) to engage in climate change communication.
Social media for social influence/as an awareness agent	Possibility for social movement on social media, collaboration via hashtags, and clear messages for social influence.

## 5. Discussion

The scoping review analyzed empirical literature on sustainability communication on social media from Asia. The purpose was to identify the direction of sustainability communication on social media in the last decade, in Asian countries. In total, 19 original research studies that met the eligibility criteria were included in this review. The methods adopted range from quantitative (n=10), qualitative (n=7), and mixed methods (n=2). Newer methods such as online ethnography point towards the growing prominence of social media-centric research ( Yang, 2022). The year range and inclusion criteria used in this study enabled the researchers to summarize the most recent findings of prevalent Asian studies on sustainability communication on social media. The theoretical frameworks of the norm activation model, value belief norm theory, uses and gratification theory and the theory of planned behavior was used in a few studies to uncover insights and guide further research ( Ch et al., 2021; Wei et al., 2021; Chen, 2020).

Broadly, the research findings of these empirical studies reveal how sustainability communication is employed by different players such as companies, causes, and the public; and how social media is used to communicate and impact behavioral change. Sustainability communication on social media in Asia is multi-directional and presents specific overarching themes.

Five prominent themes that emerged during the analysis are ‘green marketing, ‘greenwashing/skepticism towards green marketing’, ‘social media as a platform for active engagement’, ‘value of user-generated content on social media, and ‘social media for social influence/as an awareness agent’. The first theme indicates an increased interest in green marketing and subsequent green purchases ( Li et al., 2022; Kapoor et al., 2022b; Ch et al., 2021). There appears to be a symbiotic relationship between the quantum of green advertisements and other forms of sustainability communication used by brands, and the growing demand for products and services that are perceived as green by consumers ( Cao et al., 2021; Luo et al., 2020). The finding is in line with studies that present green advertisement as a powerful tool to increase environmentally responsible action such as green purchases ( Nadanyiova et al., 2020; Bailey et al., 2018). Studies on brand-generated sustainability communication focussed on customer loyalty, e-WOM, brand equity, and purchase intent ( Kong et al., 2020; Wei, et al., 2021). Wei et al. (2021) has to come before Kong et al. (2020).

Misleading claims in green advertising, consumer skepticism, and impact on purchase intent are the other directions of brand sustainability communication ( Kwon et al., 2023; Luo et al., 2020) and the second dominant theme. Interestingly, the study by Kwon et al. (2023) found that even though the number of misleading green claims by brands has increased in the past few years, the direct effect of greenwashing skepticism on purchase was not discernible. This finding contrasts with a few previous studies that found a negative impact of greenwashing on purchase intention ( Braga et al., 2019; Akturan, 2018).

The third theme presents social media as a platform for active engagement. Study findings on cause-generated sustainability communication emphasize the scope of social media especially Instagram and Facebook as effective platforms for community collaboration and to deliver campaigns for social change ( Briandana & Mohamad Saleh, 2022; Noviyanti et al., 2022; León et al., 2021; Shafqat & Fong, 2020). Studies on environmental awareness among youth present visual campaigns on Instagram, Facebook, and YouTube as high-exposure platforms for climate change information ( Chai et al., 2022; Sulistyawati et al., 2019). The fourth theme intersects with studies on user-generated sustainability communication indicating that the public is motivated by both self-presentation as well as altruistic reasons, to participate in sustainability-related discussions ( Chen, 2020; Jiang et al., 2018). The users discuss scientific and grassroots climate-related topics, with social media facilitating a participatory culture ( Yang, 2022). Testimonials on social media as a means of user-generated content are found to be more credible and effective in developing awareness as well as participation in sustainability and CSR initiatives ( Loureiro & Lopes, 2019).

The final theme that emerged highlights social media as an agent of change. Different studies have found communication of sustainability performing a key role in shaping the pro-environmental decisions of the consumers and community ( Nadanyiova et al., 2020; Bailey et al., 2018), with media, especially social media acting as the catalyst ( Yamane & Kaneko, 2021; Han & Cheng, 2020; Xu & Han, 2019). The findings of the scoping review support these conclusions and add that social media has become an important forum for climate change discussion for both scientific and non-scientific communities, for environmentalists as well as skeptics in Asia. The studies analyzed affirm that sustainability communication on social media has substantial potential to improve the environmental awareness of the public and promote actual pro-environmental behavior both in terms of green purchase as well as conservation behavior *.*

## 6. Limitations and Conclusion

The review has its limitations. Only English-language articles were considered, and the scope was restricted to research in Asia – both researchers and respondents from Asia. Future research can look at perspectives in non-English articles; also, it would be interesting to consider contributions from African and South American researchers to counterpoise American and European narratives. Further, a study could also be done on Asian researchers studying non-Asian respondents. Another limitation is that only Scopus was considered for the scoping review with the rationale that it is a comprehensive database for social science journals, however, future research can look at other databases such as JSTOR to expand the scope of the review.

The findings of the scoping review suggest that sustainability communication on social media has substantial potential to improve the environmental awareness of the public and promote actual pro-environmental behavior both in terms of green purchase as well as conservation behavior. Asian research perspectives on sustainability communication on social media are evolving; with a growing understanding of its scope, this area of research will move from a nascent stage to maturity. Moreover, with social media providing newer avenues to increase reach among newer audiences, future researchers can conduct reviews to scope the maturing levels of sustainability communication on social media. The study provides a holistic view of the recent trends and possible future directions for sustainability communication on social media. The insights presented here can help in undertaking a systematic review of the literature on sustainability communication on social media in the future.

## Ethical consideration

This marketing study was exempt from submission to the Institutional Ethics Committee of Manipal Academy of Higher Education, by clause 6 of the circular titled “Project Exemption from Submission to IEC,” issued by the Institutional Ethics Committee on 14/01/2021. Subject to this circular, approval is obtained from the MAHE PhD protocol committee (MAHE/CDS/PHD/2022) dated March 31, 2022.

Corresponding author: Dr. Smitha Nayak ( smithanayak.v@manipal.edu).

## Data Availability

The authors confirm that the data supporting the findings of this study are available within the article and its supplementary materials. The data extraction sheet with details of the research papers reviewed in this article has been uploaded on figshare. Figshare: Sustainability Communication on Social Media. Doi: https://doi.org/10.6084/m9.figshare.27314694.v1. Shetty, S (2024). This project contains the following underlying data:
• Scoping review data sheet_Shetty_Nayak.xlsx Data are available under the terms of the Creative Commons Attribution 4.0 International license (CC-BY 4.0).
