# Supplementary material for: Sustainability communication on social media in the last decade: A review of research perspectives from Asia
Source: F1000Res. 2025 Mar 19;13:1558. Originally published 2024 Dec 24. [Version 2] doi: 10.12688/f1000research.159108.2 (PMC12355160; doi:10.12688/f1000research.159108.2)
Supplement: Peer review history

# Reviewer report: Reviewer response for version 2

**DOI:** 10.5256/f1000research.179115.r391460

**Published:** 2025-08-14

**Authors:** Abu Bashar

**Affiliations:** 1 Gulf University, Sanad, Central Governorate, Bahrain

Structural and Clarity Issues

**Abstract Structure**: The abstract reads more like an introduction than a concise summary. Consider restructuring to clearly present: background, objective, methodology, key findings, and implications in a more balanced way.

**Lengthy Opening**: The first sentence is overly long and packed with multiple concepts. Break it into shorter, more focused sentences for better readability.

 Methodological Concerns

**Vague Methodology**: The phrase "comprehensive search" and "clustered around common directions" lacks specificity. Include concrete details about search terms, inclusion/exclusion criteria, and the analytical framework used.

**Unclear Scope Definition**: The definition of "Asian perspective" needs clarification. Does this mean studies by Asian researchers, studies conducted in Asia, or studies focusing on Asian populations? The current phrasing suggests all three, which may be too broad.

 Content and Language Issues

**Redundant Phrasing**: Several phrases are repetitive (e.g., "sustainability communication" appears multiple times). Vary the language or use abbreviations after first mention.

**Grammatical Errors**: "Few prominent themes" should be "A few prominent themes" or "Several prominent themes."

**Imprecise Language**: Terms like "substantial potential" and "holistic view" are vague. Use more specific language to describe the actual contributions and findings.

 Missing Critical Elements

**Gap Identification**: The abstract doesn't clearly articulate what gap in knowledge this review addresses or how it differs from previous reviews.

**Practical Implications**: While mentioned briefly, the practical implications for practitioners, policymakers, or future researchers could be more explicitly stated.

**Limitations**: Consider briefly acknowledging the study's limitations, such as the focus on one database or the geographic scope.

 Recommendations for Improvement

 Reorganize to follow a clearer abstract structure, specify the exact methodology used, define the scope more precisely, and ensure each sentence adds distinct value to the overall narrative.

Is the review written in accessible language?

Yes

Are all factual statements correct and adequately supported by citations?

Yes

Are the conclusions drawn appropriate in the context of the current research literature?

Yes

Is the topic of the review discussed comprehensively in the context of the current literature?

Yes

Reviewer Expertise:

Marketing Communications

I confirm that I have read this submission and believe that I have an appropriate level of expertise to confirm that it is of an acceptable scientific standard.

# Reviewer report: Reviewer response for version 2

**DOI:** 10.5256/f1000research.179115.r371696

**Published:** 2025-03-21

**Authors:** Sara Sousa

**Affiliations:** 1 Research Center for Natural Resources, Environment and Society (CERNAS), Polytechnic University of Coimbra, Coimbra, Portugal

Thank you for all the improvements you have made.

Is the review written in accessible language?

Yes

Are all factual statements correct and adequately supported by citations?

Partly

Are the conclusions drawn appropriate in the context of the current research literature?

Yes

Is the topic of the review discussed comprehensively in the context of the current literature?

Partly

Reviewer Expertise:

Environmental economics, pro-environmental behavior, economic valuation of environmental impacts, renewable energy sources

I confirm that I have read this submission and believe that I have an appropriate level of expertise to confirm that it is of an acceptable scientific standard.

# Reviewer report: Reviewer response for version 1

**DOI:** 10.5256/f1000research.174791.r367103

**Published:** 2025-03-04

**Authors:** Fauziyah Nur Jamal

**Affiliations:** 1 Universitas Ahmad Dahlan, Yogyakarta, Special Region of Yogyakarta, Indonesia

I have read it and the topic is very interesting, This is a good study. However, there are some weaknesses which need to be addressed further

 Abstract: Author(s) should understand that the abstract is a short note that expresses the work's contents. its contain : explored,examined,participants,surveys,measurement,result and implications. explained in more depth

 Abstract : Additionally, the literature points to the increasing usage of social media in the communication of sustainability with an increase of interest in this area among Asian researchers….Change the wording : Additionally, the literature points to the increasing usage of social media in communicating sustainability with an increase of interest in this area among Asian researchers.

 1. The introduction must be accompanied by main results on a global scale, especially current world environmental issues that encourage sustainability which can be added by looking for the latest references.

 2. Literature Review: This is fine. This could be addressed and brought to the introduction.

 3. Methodology/Materials: Please describe experimental design, data sources, research methods, and other factors that influence interpretation of model in details.

 4 Results and Findings: Good.

 5. Discussion: good. However, it could be better to see how and why this study is better than other related and recent studies.

 6. Conclusion:  practical contributions are needed in this regard.

Is the review written in accessible language?

Partly

Are all factual statements correct and adequately supported by citations?

Yes

Are the conclusions drawn appropriate in the context of the current research literature?

Yes

Is the topic of the review discussed comprehensively in the context of the current literature?

Yes

Reviewer Expertise:

sustainability, green marketing,technopreneurship

I confirm that I have read this submission and believe that I have an appropriate level of expertise to confirm that it is of an acceptable scientific standard, however I have significant reservations, as outlined above.

# Response

**Published:** 2025-03-08

**Authors:** Shruthi V Shetty

**Affiliations:** Manipal Institute of Comm., Manipal Academy of Higher Education, Manipal, Karnataka, India

Dear Ms. Jamal

 Thank you for reviewing the article and suggesting improvements. We have incorporated the suggestions and uploaded the new version of the manuscript. The response to the comments is listed below. Regards.

 Comment 1. Abstract: Author(s) should understand that the abstract is a short note that expresses the work's contents. its contain : explored,examined,participants,surveys,measurement,result and implications. explained in more depth

 Response: Thank you for the recommendation. The following additions have been made to improve text.

*Based on the analysis, the study presents the current direction of research in sustainability communication on social media in the areas of* *brand-generated sustainability communication, cause-generated content and user-generated content. Few prominent themes that emerged were of green marketing, greenwashing, s* *ocial media as a platform for active engagement* *and environment related discussions.* *The studies analyzed in the scoping review affirm that sustainability communication on social media in Asia has substantial potential to improve the environmental awareness of the public and promote actual pro-environmental behavior in the broad areas of green purchase and conservation behavior.*

 Comment 2. Abstract : Additionally, the literature points to the increasing usage of social media in the communication of sustainability with an increase of interest in this area among Asian researchers….Change the wording : Additionally, the literature points to the increasing usage of social media in communicating sustainability with an increase of interest in this area among Asian researchers.

 Response: We acknowledge the suggestion. The necessary change in wording has been completed.

 Comment 3. The introduction must be accompanied by main results on a global scale, especially current world environmental issues that encourage sustainability which can be added by looking for the latest references.

 Response: We appreciate the recommendation. The following additions have been made to improve text and additional references have been incorporated.

*Globally, there is a growing awareness about, the imminent catastrophe due to environmental degradation as well as the steps necessary to safeguard ecology ( Crapa et al., 2023; Camilleri, 2017). Issues such as extreme weather conditions (Chen et al., 2023), extinction of species (Souza et al., 2022), imbalance in biodiversity (Corriea et al., 2023) and increased greenhouse gas emissions (Higueras‐Castillo et al., 2024) are of serious concern.*

*The UN Agenda 2030 for Sustainable Development aims towards accomplishment of a balanced economic, social and environmental development. The United Nations Brundtland Commission (1987) defined sustainability as meeting the needs of the present without compromising the ability of future generations to meet their own needs. Sustainability is a buzzword today, subsumimg other often used terms such as triple bottom line, sustainable development, green business, corporate citizenship and corporate social responsibility (Kumar & Christodoulopoulou, 2013; Reilly & Larya, 2018).*

*Green marketing communication has emerged as one of the leading ways in which corporates present their stance on sustainability as this fuels green purchase and proves profitable for them. (Corriea et al., 2023; Jamal et al., 2022; Jamal et al., 2021).*

 Comment 4. Literature Review: This is fine. This could be addressed and brought to the introduction.

 Response: We acknowledge the suggestion. After deliberation, we found it more appropriate to retain the existing structure. The objective of this research endeavour was to review the literature published in the domain of sustainability communication on social media. Therefore, the introduction section of the manuscript captures the review of literature connected to the subject. The introduction has been strengthened with more references.

 Comment 5. Methodology/Materials: Please describe experimental design, data sources, research methods, and other factors that influence interpretation of model in details.

 Response: Thank you for the suggestion. In total 19 research studies have been included in the study. Details of the data sources has been highlighted in 2.1. Search Strategy. Additional figures have been added to elucidate more details of the selected studies under 2.4. Data Extraction. Figure 2. presents the types of research designs adopted in the reviewed papers. Figure 3. exhibits a network graph of the keywords used in the included articles.

 The details of each of the papers included in the study is presented in Table 1. Summary of articles included in the study

 Comment 6. Results and Findings: Good.

 Comment 7. Discussion: good. However, it could be better to see how and why this study is better than other related and recent studies.

 We acknowledge the suggestion. The following section has been added to the discussion and conclusion segment to address this.

*Some notable points that emerged from the scoping review needs added emphasis. The role of social media influencers is being deliberated by industry professionals and researchers. In sustainability communications, green influencers and their content has the potential to be a persuasive voice of change. The pro-environmental behavior in the reviewed studies can be grouped into two main types, green purchase behavior that includes buying products that are labelled as green, and conservation behaviour such as knowledge-sharing, cause awareness and working toward protection of the environment. Sustainability communication on social media influences both the categories of PEB among Asian respondents. The influence of social media on youth or Gen Z is overarching and is true for communication of sustainability. Visual communication is seen as more effective than textual communication and it can be deduced that the effect of social media is platform agnostic; currently Instagram and YouTube take precedence, in the future any other visual-first medium may have the ability to influence pro-environmental behaviour. Social media with its participatory culture, is also evolving as an important forum for climate change discussion for both scientific and non-scientific communities, for environmentalists as well as skeptics in Asia.*

 Comment 8. Conclusion:  practical contributions are needed in this regard.

 Thank you for the suggestion. The content has been reframed to highlight the practical contribution so the paper.

*The findings of the scoping review suggest that sustainability communication on social media has substantial potential to improve the environmental awareness of the public and promote actual pro-environmental behavior in the two significant fields of green and conservation behavior. The study findings can be useful for researchers as it lists the current focus areas. The unique contribution of the paper is its emphasis on the Asian research perspectives on sustainability communication on social media which is evolving; with a growing understanding of its scope, this area of research will move from a nascent stage to maturity.*

# Reviewer report: Reviewer response for version 1

**DOI:** 10.5256/f1000research.174791.r368268

**Published:** 2025-02-27

**Authors:** Sara Sousa

**Affiliations:** 1 Research Center for Natural Resources, Environment and Society (CERNAS), Polytechnic University of Coimbra, Coimbra, Portugal

The research paper is interesting, but needs some improvements, namely:

- In the introduction, authors should add more references and improve the text.
- Also in the introduction, authors should give the definition of the main concepts, such as sustainable development, sustainability, among others.
- I also suggest the authors to improve the text…it seems you write about  different key subjects, but you do not go deeper.
- I suggest authors to read the work of Correia et al. and Sousa et al. These authors have published interesting papers on this subject
- In section 3 of data analysis, authors should develop more the subject. I am sure there is much more to write on this subject
- I also advice authors to enrich the paper with image, there is many interesting ways to do it, namely with clouds of keywords, etc.
- And the discussion needs to be improved

Is the review written in accessible language?

Yes

Are all factual statements correct and adequately supported by citations?

Partly

Are the conclusions drawn appropriate in the context of the current research literature?

Yes

Is the topic of the review discussed comprehensively in the context of the current literature?

Partly

Reviewer Expertise:

Environmental economics, pro-environmental behavior, economic valuation of environmental impacts, renewable energy sources

I confirm that I have read this submission and believe that I have an appropriate level of expertise to confirm that it is of an acceptable scientific standard, however I have significant reservations, as outlined above.

# Response

**Published:** 2025-03-08

**Authors:** Shruthi V Shetty

**Affiliations:** Manipal Institute of Comm., Manipal Academy of Higher Education, Manipal, Karnataka, India

Dear Ms. Sousa

 Thank you for reviewing the article and suggesting improvements. We have incorporated the suggestions and uploaded the new version of the manuscript. The response to the comments is listed below. Regards.

**Comment 1.** In the introduction, authors should add more references and improve the text.

 Response: Thank you for the recommendation. The following additions have been made to improve text and additional references have been incorporated. The corresponding citations has been included in the references.

*Globally, there is a growing awareness about, the imminent catastrophe due to environmental degradation as well as the steps necessary to safeguard ecology ( Crapa et al., 2023; Camilleri, 2017). Issues such as extreme weather conditions (Chen et al., 2023), extinction of species (Souza et al., 2022), imbalance in biodiversity (Corriea et al., 2023) and increased greenhouse gas emissions (Higueras‐Castillo et al., 2024) are of serious concern.*

*…a difference can also be made by undertaking micro-level change in the form of behavior interventions (Stieglitz et al., 2023) on people who consume materials and energy every day.*

 Few more added in the relevant places of the manuscript

**Comment 2. **Also in the introduction, authors should give the definition of the main concepts, such as sustainable development, sustainability, among others.

 Response: We acknowledge the suggestion; the following section has been added to the introduction.

*The UN Agenda 2030 for Sustainable Development aims towards accomplishment of a balanced economic, social and environmental development. The United Nations Brundtland Commission (1987) defined sustainability as meeting the needs of the present without compromising the ability of future generations to meet their own needs. Sustainability is a buzzword today, subsumimg other often used terms such as triple bottom line, sustainable development, green business, corporate citizenship and corporate social responsibility (Kumar & Christodoulopoulou, 2013; Reilly & Larya, 2018).*

**Comment 3. **I also suggest the authors to improve the text…it seems you write about  different key subjects, but you do not go deeper.

 Response: Thank you for the suggestion. Few improvements/additions in the following sections have been carried out - Introduction, Results and the Discussion section.

**Comment 4.** I suggest authors to read the work of Correia et al. and Sousa et al. These authors have published interesting papers on this subject

 Response: We appreciate the suggestion; studies by Correia et al. and Sousa et al. were referred and included in the relevant segments – 1. Introduction and 4.1. Theoretical Underpinnings. The corresponding citations has been included in the references.

*Green marketing communication has emerged as one of the leading ways in which corporates present their stance on sustainability as this fuels green purchase and proves profitable for them.* *(* *Corriea et al., 2023; Jamal et al., 2022; Jamal et al., 2021).*

*Azjen’s Theory of Planned Behaviour (TPB) is largely used by researchers to predict pro-environmental behavior; the core components of subjective norms, perceived behavioral control, and specifically attitude in varying degrees correlate to environmentally significant behavior ( Corriea et al., 2022; Souza et al., 2022; Yang et al., 2020; Paul et al., 2016; Ho et al., 2014).*

**Comment 5:** In section 3 of data analysis, authors should develop more the subject. I am sure there is much more to write on this subject

 Response: We acknowledge the suggestion; few more points have been added to the data analysis section.

- *The researchers conducted a detailed analysis of the selected 19 studies, with careful deliberation over each of the study particulars mentioned in Table 1. To provide a coherent structure to the results, the insights were categorized into the following segments: theoretical frameworks, the direction of sustainability communication on social media in Asia, utility of social media in sustainability communication, and the prominent themes of sustainability communication on social media in Asia.*

**Comment 6.** I also advice authors to enrich the paper with image, there is many interesting ways to do it, namely with clouds of keywords, etc.

 Response: We appreciate the recommendation; two images have been added accordingly. Figure 2. presents the types of research design adopted in the reviewed papers. Figure 3. exhibits a network graph of the keywords used in the included articles.

**Comment 7.** And the discussion needs to be improved

 Thank you for the suggestion. The following section has been added to the discussion segment.

*Some notable points that emerged from the scoping review needs added emphasis. The role of social media influencers is being deliberated by industry professionals and researchers. In sustainability communications, green influencers and their content has the potential to be a persuasive voice of change. The pro-environmental behavior in the reviewed studies can be grouped into two main types, green purchase behavior that includes buying products that are labelled as green, and conservation behaviour such as knowledge-sharing, cause awareness and working toward protection of the environment. Sustainability communication on social media influences both the categories of PEB among Asian respondents. The influence of social media on youth or Gen Z is overarching and is true for communication of sustainability. Visual communication is seen as more effective than textual communication and it can be deduced that the effect of social media is platform agnostic; currently Instagram and YouTube take precedence, in the future any other visual-first medium may have the ability to influence pro-environmental behaviour. Social media with its participatory culture, is also evolving as an important forum for climate change discussion for both scientific and non-scientific communities, for environmentalists as well as skeptics in Asia*.
